# Stochastic Responses May Allow Genetically Diverse Cell Populations to Optimize Performance with Simpler Signaling Networks

**DOI:** 10.1371/journal.pone.0065086

**Published:** 2013-08-07

**Authors:** Christopher C. Govern, Arup K. Chakraborty

**Affiliations:** 1 Department of Chemical Engineering, MIT, Cambridge, Massachusetts, United States of America; 2 Department of Chemistry, MIT, Cambridge, Massachusetts, United States of America; 3 Department of Biological Engineering, MIT, Cambridge, Massachusetts, United States of America; 4 Ragon Institute of MGH, MIT, & Harvard, Charlestown, Massachusetts, United States of America; Texas A&M University, United States of America

## Abstract

Two theories have emerged for the role that stochasticity plays in biological responses: first, that it degrades biological responses, so the performance of biological signaling machinery could be improved by increasing molecular copy numbers of key proteins; second, that it enhances biological performance, by enabling diversification of population-level responses. Using T cell biology as an example, we demonstrate that these roles for stochastic responses are not sufficient to understand experimental observations of stochastic response in complex biological systems that utilize environmental and genetic diversity to make cooperative responses. We propose a new role for stochastic responses in biology: they enable populations to make complex responses with simpler biochemical signaling machinery than would be required in the absence of stochasticity. Thus, the evolution of stochastic responses may be linked to the evolvability of different signaling machineries.

## Introduction

Stochastic cellular responses have been observed in varied biological contexts [1]–[4]. They are sometimes inferior approximations to deterministic responses [1], [5], [6], caused by noise in biochemical reactions. But they are not always inferior. For example, stochastic phenotype selection can help isogenic, sensorless populations of bacteria survive in varying environments [7]–[12]. The stochastic, and therefore diverse, phenotypes reduce the population's risk of extinction as the environment cycles through states that are adverse to individual phenotypes. In general, stochastic diversification is known to benefit various populations that are isogenic and sensorless, or modeled as such [7]–[15]. Stochastic responses are the only way for these populations to diversify their responses, which can be beneficial for system-specific reasons.

Many important biological systems are genetically (epigenetically) diverse, or have sensors for diverse environments. Cells in such populations can exploit the differences in their genotypes or in their receptor inputs to make diverse responses, making stochasticity unnecessary. However, if genetic or environmental diversity is limited (e.g. 99% of the cells are isogenic), stochastic responses may be required to enhance diversification (e.g. obtain a 50–50 phenotypic split). Thus, Wolf et al. have demonstrated that stochastic responses can optimize growth rate in bacterial populations able to sense, with error, only a limited number of different environmental states, even though the added noise corrupts the information received through the sensors [12].

The role of stochastic responses in populations which utilize considerable environmental or genetic diversity to diversify their responses is less understood [1]. We use T cells, key orchestrators of the adaptive immunity, as an important example in order to consider the role of stochastic responses in such systems. Each T cell has a receptor (or sensor), the T cell receptor (TCR), and most T cells express a unique TCR. These different receptors bind with varying strengths (e.g. affinity) to diverse peptides (p), derived from pathogenic and self proteins, which are expressed on antigen-presenting cells (APCs) in complex with host major histocompatibility (MHC) proteins [16]. TCRs tend to bind self-derived peptides weakly due to a developmental process, thymic selection. T cells bearing TCR that bind strongly to self-pMHC in the thymus are likely deleted from the host repertoire [17]. Consequently, the strength of the interaction between a T cell's receptors and the pMHCs presented on an APC provides information to a T cell about whether at least some of the pMHCs are pathogen-derived, so the T cell should respond to clear infection, or whether they are all self-derived, so the T cell should remain inactive to prevent autoimmunity. Specifically, strong binding indicates an interaction with pathogenic pMHC. Weak or intermediate binding is less conclusive because thymic selection is imperfect and because some pathogens exhibit peptides that bind relatively weakly to TCR.

Thus, T cells are an example of a population that utilizes genetic (receptor) and environmental (diverse stimuli) diversity to make cooperative responses within a host. It would appear that T cells do not require stochasticity to diversify their responses. However, over a range of TCR-pMHC binding affinity, or strength of other stimuli such as that provided by cytokines, some T cells fire and others do not, due to intrinsic stochasticity in the T cell's molecular signaling machinery and/or to external noise [18]–[21]. Is the stochastic response of this important system just “noise”? Consistent with the considerable genetic and environmental diversity T cells utilize, the results of a mathematical model suggest that it is [22].

However, by studying a model of T cells that captures complex ways they interact with each other and their environment, we find that their stochastic responses are not necessarily “noise.” The environmental and genetic diversity available to T cells is sufficient for them to make diverse responses, but the signaling machinery required to implement these diverse responses deterministically is exceedingly complex. We find that stochastic responses can enable populations like T cells to achieve similar performance with relatively simple signaling machinery. Thus, biological populations that utilize considerable environmental and genetic diversity may benefit from stochasticity because of limitations in biochemical signaling machinery, not because stochasticity is necessary for diversification or optimal performance, as in isogenic, sensorless populations.

## Materials and Methods

To study responses based on environmental and genetic diversity, we consider a model of T cell interactions and their outcomes which abstracts general features observed in experiments. Motivated by experiment, we focus on naive T cells and their decisions to activate, as opposed to other T cell subsets or lineage commitment decisions. The naïve T cells scan APCs that may or may not present pathogenic pMHC. In each encounter with an APC, a T cell makes a stable binary decision to either activate or not [18], [23]. These decisions are determined by the T cell's intracellular signaling machinery based on inputs from the many receptors on the T cell's surface, including TCR and cytokine receptors. For now, we focus on inputs to the T cell from the many (identical) TCRs, which engage peptides on an APC's surface with varying affinities during the course of an APC-T cell interaction; we discuss cytokine signals later. For clarity, we summarize the many inputs from individual TCRs to the T cell's signaling network with a single stimulus strength, *x*, which may represent the concentration of a membrane proximal signaling molecule that integrates the input of all bound TCR [6].

**Figure 1:**
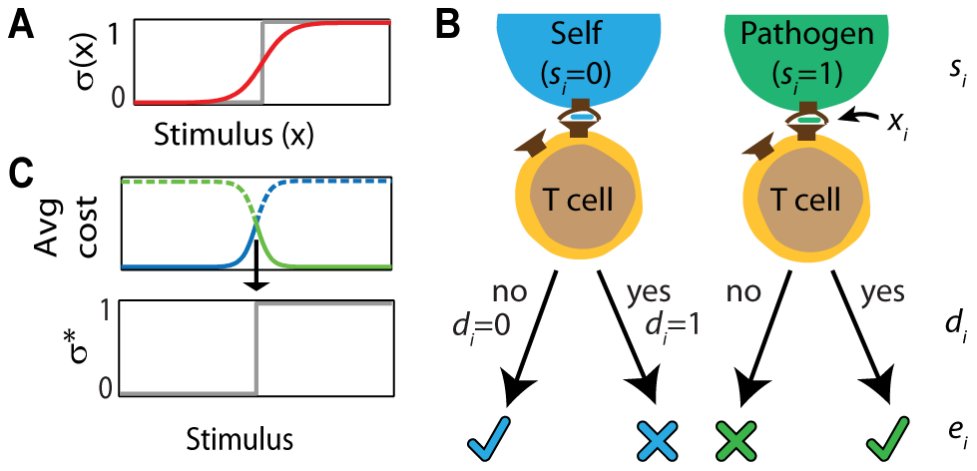
T cells make stochastic decisions. (A) A T cell's activation probability,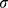, is governed by a stochastic decision rule (red), not a sharp deterministic threshold (grey; [18]–[21]. (B) The variable *s_i_* denotes whether the interaction is with self or pathogenic pMHC; *x_i_* is the stimulus strength (e.g. TCR-pMHC binding strength); *d_i_* is the actual decision made (yes, activate; no, remain inactive); and *e_i_* specifies whether the decision is correct (check) or not (x) against self (blue) or pathogenic (green) pMHC (four possibilities). (C) An isolated T cell should activate whenever the expected or average cost of not activating (e.g. blue) is greater than the expected cost of activating (green), corresponding to an optimal deterministic decision rule,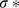, where activation occurs above a sharp threshold stimulus strength.

T cells' decisions, based on inputs to their signaling network, are observed to be stochastic: the probability of activation (σ(x) in Fig. 1A) increases from approximately zero to approximately one over a finite range of stimulus strengths [18], [23]. For example, the Ras-SOS signaling pathway, a critical T cell signaling pathway, exhibits this non-deterministic behavior. We refer to the probabilities of activation σ(x), determined by the intracellular signaling machinery, as the T cells' decision rule. The T cells' stochastic decision rule contrasts to deterministic decision rules, for which the probabilities of activation σ(x) are always either 0 or 1. The particular deterministic decision rule T cells would obtain by suppressing stochasticity in their signaling machinery is a simple sharp threshold, which prescribes activation whenever the stimulus is strong enough (Fig. 1b; [18]).

How do these different decision rules compare in terms of the ultimate outcome for a host? The decisions made according to a decision rule influence outcomes in two ways: 1] Certain outcomes, like autoimmunity and persistent infection, depend on whether the decisions are correct or not in response to APCs bearing only self or also pathogenic pMHC. If too many T cells activate upon interactions with self pMHC, autoimmunity would ensue. If too few T cells activate in response to a pathogen's pMHC molecules, persistent infection or death could result. 2] Other outcomes depend only on which decision is made, regardless of whether it is correct. For example, a decision to activate incurs a metabolic cost.

To compare qualitatively dissimilar outcomes (e.g. infection and autoimmunity), we quantify outcomes by a cost *C*. Then, one decision rule is better than another if it would lead, on average, to outcomes with a lower cost to the host, as quantified by the expected cost, *E[C]* [24], [25]. As in isogenic, sensorless populations that have been studied (e.g. [9]), it is necessary to compare average outcomes. Diverse stochastic processes intrinsic to an immune response make the exact outcome for the host variable: two hosts may have different success clearing infection and avoiding autoimmunity even though their T cells make decisions according to the same decision rule (i.e. have the same intracellular signaling machinery).

We express the expected cost in terms of quantities directly related to the decision rule: the stimulus strengths a population of T cells receive through their receptors during an infection (**x**), the decisions (**d**) they make based on them, either to activate (*d_i_* = 1) or remain inactive (*d_i_* = 0), and whether these decisions are correct or errors (**e**) (Fig. 1c). Note that **x**, **d**, and **e** are all vectors. Each element of the vectors corresponds to the stimulus strength, decision, and error in an individual T cell-APC interaction during the course of an infection. The number of interactions during the course of an infection, *N*, may itself be stochastic. Then, the expected cost can be written as:
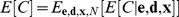
(1)


The inner expectation is independent of the decision rule. It quantifies how the outcome, on average, depends on the actual decisions that are made (e.g. metabolic costs) and whether or not they are errors (e.g. infection and autoimmunity), as described above. We assume below that the outcome does not depend directly on the stimuli**x**, writing the inner expectation as *E[C|* ***e*** *,* ***d*** *]*. The outer expectation is taken over the joint variability of the decisions and errors that are made and the stimuli that are seen (**d**, **e**, and **x**), which is described by a probability distribution, *P(* ***e*** *,* ***x*** *,* ***d*** *,N)*. The specific form of this probability distribution is partly determined by the decision rule: by definition, the decision rule is the probability of activation (*d_i_ = 1*) given a stimulus strength *x_i_*, *P(d_i_ = 1|x_i_)*. Detailed work on stochastic effects in the immune system, separate from the stochasticity of the T cell response itself, suggests other features of the probability model that we describe later. For example, detailed models of the interaction between T cells and APCs suggest certain features of probability models for the stimulus strength, *x_i_* [6].

For clarity, we introduce **s**, the APC type, a vector that lists whether each interaction is with an APC bearing only self- (*s_i_* = 0) or also pathogenic- (*s_i_* = 1) pMHC. Then, whether a T cell makes an error, *e_i_,* can be determined by comparing its decision *d_i_* to the correct decision, determined by the APC type *s_i_* (if an APC bears pathogenic pMHC, *s_i_* = 1, then the correct decision is to activate.) We refer to the APC type, *s_i_,* and the resultant stimulus strength, *x_i_,* as jointly specifying the T cell's “encounter.” In terms of this notation, the expected cost can be rewritten as:
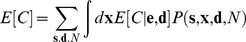
(2)where we have expanded the outer expectation in Eq. 1.

Eq. 2 provides a general framework to which biological detail can be added to compare the performance, *E[C]*, of stochastic and deterministic decision rules. Although we have motivated Eq. 2 by describing T cell biology, its form is general enough to consider decisions by cooperative populations utilizing environmental or genetic diversity.

This framework has in common with canonical frameworks in statistics and decision theory that all decision-makers or players have a single payoff function (cost) and their interactions may be uncorrelated. It has in common with game theory that decision-makers do not necessarily share the same information or communicate it fully, and they can make different decisions. It has in common with sequential decision theory (e.g. dynamic programming) that *P(* ***s*** *,* ***x*** *,* ***d*** *,N)* may describe a process. Stochastic decisions at the population-level are familiar in these larger contexts (see [24] for stochastic decision making in sequential decision theory; and [26] for a discussion in game theory). In these contexts, the results below illustrate how stochastic responses can emerge under the particular conditions relevant to biological systems.

## Results

### A stochastic decision rule can outperform simple deterministic decision rules, but only in strongly coupled populations

We first compare T cells' stochastic responses to the relatively simple deterministic decision rule experiments suggest they would obtain by suppressing noise in their signaling machinery, a deterministic sharp threshold [18]. We consider several models of increasing complexity. If the stochastic response outperforms the simple deterministic response even for a simple model, we reason that this will also be the case for more complicated models, as explained below.

#### A simple deterministic decision rule optimizes the response of isolated T cells

Consider the simplest case of an isolated T cell in a single interaction (*x*, *e*, *d*, and *s* are now scalars). For a given stimulus strength, *x*, the expected cost *E[C|x]* is the expected cost of activating,*E[C|x,d = 1]*, weighted by the probability of activation (σ(x)), and the expected cost of not activating, *E[C|x,d = 0]*, weighted by the probability of not activating (1-σ(x)):
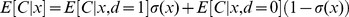
(3)


Note that *d* = 1 and *d* = 0 correspond to decisions to activate and remain inactive, respectively.

Because of thymic selection, self-peptides are more likely to stimulate T cells weakly than strongly, as noted above [6], [17]. Therefore, the expected cost of activating for very weak stimuli is higher than for very strong stimuli. For simplicity, we initially assume the expected cost for activation is a strictly decreasing function of the stimulus strength *x*, whereas the expected cost of not activating increases with *x.* As illustrated in Fig. 1C, the expected cost for not activating then exceeds that of activating at a single stimulus strength. Eq. 3 shows that the choice of the decision rule σ that minimizes the expected cost is σ = 0 (never activate) if the expected cost of activation exceeds the expected cost of not activating, and σ = 1 (always activate) if the opposite is true. Therefore, the optimal decision rule for isolated T cells is a deterministic sharp switch from not activating to activating and could be implemented by the existing T cell signaling machinery if noise was suppressed (e.g., with more molecules). The same conclusion holds even if the expected costs are not strictly increasing (decreasing), as long as they do not intersect more than once. Why do T cells not suppress this “noise”?.

#### T cells are coupled at the population level

T cells, like other cells that make population-level responses, do not act in isolation. Function is determined by the response of the entire population, interacting with each other and the host to produce an outcome. We use T cell biology to motivate the qualitatively different ways in which individual cells can be coupled to others in a population.

**Figure 2:**
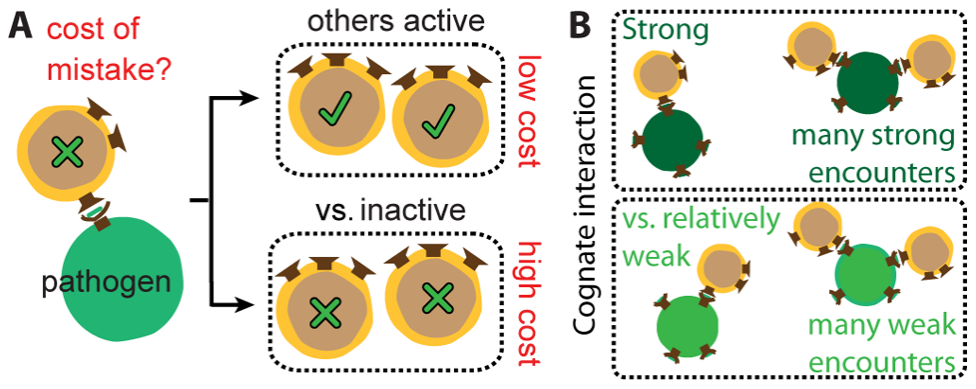
T cells are coupled at the population level. (A) T cell decisions are coupled through the common outcome for the host. For example, the cost of a T cell mistakenly not activating (green x) against a pathogenic pMHC depends on the correctness of other T cells' decisions. If enough others activate (green check) in response to this pathogen, the mistake has minimal impact since the infection will be cleared (low cost). Conversely, if other T cells have not been activated, there is a high cost for the T cell not activating as the pathogen will proliferate unchecked. (B) T cell decisions are coupled because they sense the same environment, residing in the same host and confronting the same infection. For example, if an infection expresses a peptide with a particularly strong cognate interaction (dark green), then all T cells of the same clonotype will receive similarly strong stimulus strengths in their interactions. If an infection expresses a peptide with a relatively weak cognate interaction (light green), then the stimulus strengths received by T cells during that infection will all be relatively weak. T cells are also coupled because they can change their environment, but this is not illustrated.

One form of coupling arises because T cells collectively contribute to the common outcome for the host. Mistakes or actions by one T cell can be exacerbated or recovered by the actions of others. In terms of the model, the cost incurred by one T cell's decision depends on the decisions of other T cells (Fig. 2A). For example, the cost of a T cell mistakenly not activating in response to a pathogenic pMHC is lower if many T cells have been activated in response to the infection since only a certain level of activation is required to clear infections. Also, the cost of activating against an APC bearing only self pMHC is higher if similar events have already occurred since peripheral tolerance mechanisms can tolerate only some autoimmune responses. The coupling of T cell responses through the common outcome means that the expected cost *C* to the host associated with the population's collective decisions **d** and errors **e** is not just the sum of costs, *C_i_*, incurred in individual interactions *i*:
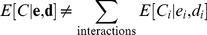
(4)


Another form of coupling arises because T cells reside in, and sense, the same environment (i.e. they are in the same host confronting the same infection.) For example, many infections present multiple immunodominant epitopes. T cells that respond to different immunodominant epitopes in an infection will receive similarly strong stimulus strengths in their interactions. Alternately, when an infection successfully suppresses expression of pathogenic peptides on APCs, all T cells are less likely to encounter a pathogen-bearing APC. Thus, the stimulus strengths different T cells receive through their receptors, **x**, are not independent (Fig. 2B), and neither are the APC types they encounter. Then, the joint probability of the encounters is not just the product of probabilities of individual encounters:
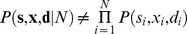
(5)


We have included the decisions, **d**, in Eq. 5, for comparison with Equation 2.

Finally, T cells are coupled because they can change their common environment. For example, if a T cell activates in response to a particular pMHC expressed upon infection by a fast – mutating virus, the resulting immune pressure will cause the outgrowth of a mutant strain that will present pMHC that may strongly stimulate another T cell. Thus, the decision of one T cell (*d_i_*) can affect the encounters (**s**, **x**) of other T cells. The two are not independent:
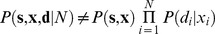
(6)


Other features of the immune system provide additional motivations for each of these types of coupling when viewed at the level of coarse-graining in our model. For example, in addition to the canonical TCR-pMHC stimulus, T cells receive cytokine stimuli that influence their responses. The model accounts generically for these cytokine stimuli by incorporating them in the stimulus strength *x* (possibly by making *x* a vector for each encounter.) Then, the encounters (e.g. strengths of a particular cytokine stimulus) of different T cells are coupled because they are in the same cytokine environment; and the decision of one T cell to activate (consuming and releasing cytokines) affects the interaction (cytokine stimulus) of other T cells.

Importantly, however, these other features of the immune system, not explicitly considered, are unlikely to decouple the T cells. We use Eqs. 4 through 6, motivated by the T cell population, to study the effects of coupling in population-level responses that utilize environmental and genetic diversity.

### A simple deterministic decision rule optimizes the performance of populations coupled only through a common outcome

As described above, T cells are coupled. We considered whether adding different forms of coupling to our model of the T cells changes the effect of suppressing noise in their signaling machinery, again comparing the T cells' stochastic response with a simple deterministic sharp threshold.

For isogenic, sensorless populations, coupling through the common outcome (population growth) is critical to the optimality of stochastic response (i.e. population growth is nonlinear) [2], [15], [27]. Therefore, we added this to the model of the T cell population (Eq. 4). However, without other forms of coupling, each T cell makes an error (activates against self or does not activate against pathogens) independently. The overall probabilities of a T cell making an error are:


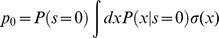
  =  probability of incorrectly activating (7a)


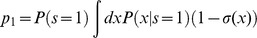
 =  probability of incorrectly not activating (7b)

The integral in Eq. 7a, 
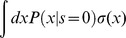
, is the probability of mistakenly activating against an APC bearing only self, calculated as the probability the stimulus strength in the encounter activates the T cell, σ(x), averaged over the probability the stimulus strength is *x* in encounters with APC bearing only self, *P(x|s = 0).* The probability of mistakenly activating is then this probability times the probability a T cell encounters an APC bearing only self, *P(s = 0).* A similar logic leads to Eq. 7b. Note that these probabilities are obtained by integrating the probability model in Eq. 2 over all other variables. The form of the probabilities in Eq. 7 is analogous to the form considered in the Neyman-Pearson lemma (e.g. type 1 and type 2 errors; [28]). The Neyman-Pearson lemma states that the decision rule jointly minimizing the probabilities of error in Eq. 7, and therefore the expected cost in Eq. 2, is a single deterministic sharp threshold, when the likelihood of one action being correct increases with the stimulus, as for T cells.

Specifically, any candidate decision rule leads to particular values of the probabilities in Eq. 7. Consider a particular candidate decision rule that is not of a single sharp threshold form. According to the Neyman-Pearson lemma, one can find a single sharp threshold decision rule that has the same probability of incorrectly not activating but a lower probability of incorrectly activating. This single sharp threshold, therefore, will have a lower cost due to errors, on average. Furthermore, the single sharp threshold has a lower probability of activating (since it activates correctly just as often, but activates incorrectly less often), and so incurs a lower cost due to resource consumption, on average. Regardless of the structure of the cost function, then, the single sharp threshold will have a lower cost, on average. Since this is true regardless of the candidate decision rule, the optimal decision rule must have the form of a single sharp threshold. The particular location of the threshold will depend on the exact form of the cost function and the probabilities.

Accordingly, optimization of the quantities (7a) and (7b) in a detailed modeled of T cell interactions is consistent with many aspects of the T cell immunology, but not their stochastic response [22].

### A simple deterministic decision rule optimizes the performance of populations coupled only through sensing a common environment

Additional types of coupling are possible in populations that utilize environmental or genetic diversity, so we considered the effect of adding one of these to the model, the fact that they sense a common environment (Eq. 5). If T cells were coupled only through residing in a common environment the inequality in Eq. 4 would not hold, and the resulting linearity makes treating the coupling through the environment easy. We find that the expected cost in Eq. 2 depends linearly on the decision rule σ(x) (see below):
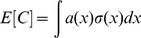
(8)where *a* is a function that depends on the probability model and cost function but not on the decision rule. Arguments analogous to those for the isolated T cell (see below) suggest that the optimal decision rule for T cells is a single sharp threshold (not stochastic).

Specifically, under coupling through the environment alone, the expected cost in Eq. 2 can be simplified. The following steps, resulting in Eq. 9, consist of simple algebraic manipulations, exploiting: (1) the linearity of the cost function (Eq. 4), so that any dependencies in the observations are integrated out in the expectation; and (2) the independence of the *i^th^* decision from all encounters other than the *i^th^* encounter, so that the decision rule σ(x) can be isolated from the probability *P(x,s)*.

First we consider the number of interactions *N* to be fixed (given). We adopt the notation:
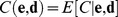



Also, let (*) denote the conditional expectation:
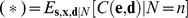



Then, using the assumed linearity of the cost function (from Eq. 4 with equality):
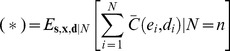
where we have written *C_i_* as 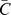 to emphasize that it depends on i only through its arguments. Bringing the expectation inside the summation (since *N* is given):



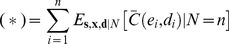



Then, because 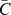 depends only on one interaction at a time (the *i^th^*), the expectations can be taken trivially over all variables not associated with the *i^th^* interaction:
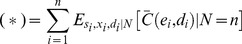



Expanding the expectation as a sum/integral over the variables *x_i_*, *s_i_*, and *d_i_*, weighted by their probabilities:
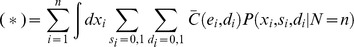



Recruiting the assumption that the encounters are independent from the total number of interactions, since the population is coupled only through its environment:
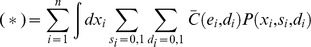



Because, by assumption, the decisions do not affect the encounters (Eq. 6 with equality, integrated over all variables but those corresponding to the *i^th^* interaction):


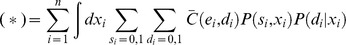
Expanding the summation over *d_i_*:
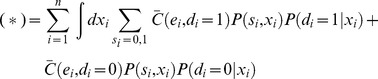



Applying the definition of σ(x):
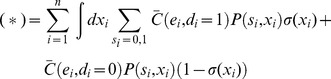



Grouping terms according to σ*(x)* and compacting the notation (the dependence on *i* comes only because the coarse-grained probability may depend on *i*):
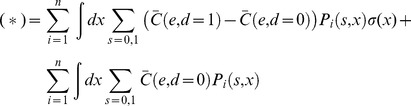



The second term does not depend on σ*(x)* and therefore does not affect the optimization over σ*(x)*. For compactness, we suppress it in what follows:
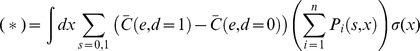



To derive these previous equations, we assumed *N* was given. This assumption can be relaxed:
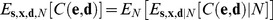



Substituting the expression that was derived for (*) into the right hand side:
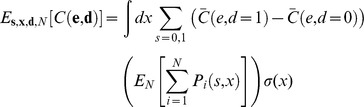



In principle, *P_i_(s,x)* can depend on *i* in two ways: through *P_i_(x|s = 0)* and *P_i_(x|s = 1)* or through *P_i_(s = 0)* and *P_i_(s = 1)*. In the following we assume that the dependence comes at most through *P_i_(s = 0)* and *P_i_(s = 1)*; that is, the stimuli from self and pathogenic pMHC come from stationary processes (in the sense that the initial conditions are also averaged over). We make this assumption because more complicated behavior in the coarse grained model would seem to implicate one of the other forms of coupling (e.g. decisions affecting observations), which we have excluded in this proof by assumption.When *P_i_* does not depend on *i*, this previous equation can be simplified to the following, which is the main result of the preceding manipulations:



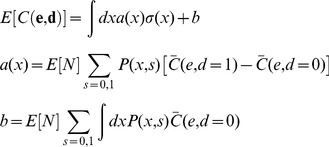
(9)When *P_i_* depends on *i,* but as above, the proof follows similarly. Recall that *e* is a function of *d* and *s*, and so is fully determined in the expressions for *a(x)* and *b* in Eq. 9.

Because Eq. 9 is a linear functional of σ(x), the optimization of σ(x) in Eq. 9 can be done at each value of *x* separately. Specifically,
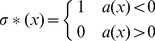
(10)


Note that for *a(x)* exactl y equal to 0, σ*(x) can take any value. We have assumed here that the set of such *x* is insignificant (e.g. a set of 0 measure.) With simple algebra, the requirement that *a* is negative corresponds to:
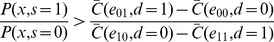
(11)where the notation *e_sd_* denotes the value of *e* when the correct decision is *s* and the actual decision is *d*. (The numerical value of *e_sd_* is arbitrary, so long as 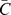 is defined consistently.) By assumption, as described for isolated T cells, the left hand side in Eq. 11 is strictly increasing with *x*. Therefore, if 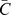
is independent of *x*, Eq. 11 corresponds to a single sharp threshold, as described for an isolated T cell. When 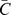 depends on *x*, it is harder to draw general conclusions. However, the best solution will still be a single sharp threshold as long as the difference in the expression for *a(x)* in Eq. 9 changes sign only once. The arguments in this section recall the Neyman-Pearson lemma (21).

Thus, stochastic optimality is not a generic feature of coupled populations, as suggested by populations which lack environmental and genetic diversity. Populations that utilize diverse information from their receptors and genome and which are only weakly coupled (only through residing in a common environment or only through a collective impact on the outcome) can benefit from suppressing noise in their signaling machinery. Strong coupling, in the ways described above, is a necessary condition for stochasticity to be useful in biological populations that are neither isogenic nor sensorless.

### Stochastic decision rules can outperform simple deterministic decision rules in strongly coupled populations

We have argued that, regardless of the specific details included in a model, T cells are not merely weakly coupled. Therefore, to understand stochasticity in populations that are strongly coupled, we considered the effect of suppressing stochasticity in a model of the T cells as a strongly coupled population. We explicitly model their collective contributions to the outcome and their common environment (Eqs. 4 and 5). Their collective contributions to the outcome are treated by noting that the cost incurred by the host over the course of a single infection decreases nonlinearly with the amount of activation against APCs bearing pathogenic pMHC and increases with the amount of activation to APCs bearing only self pMHC. Thus the average cost *C* associated with the T cell errors **e** and decisions **d** is:
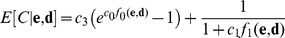
(12)which satisfies Eq. 4, where *f_0_* and *f_1_* denote the fractions of APCs bearing only self or also pathogenic pMHC to which T cells activate, respectively; these fractions are determined by the decisions **d** and the errors **e**. Our qualitative conclusions do not depend on specific nonlinear form of Eq. 12 or the particular values of the constants *c_1_*, *c_2_*, and *c_3_*, which weight the cost of activation against APCs bearing only self-pMHC against the cost of failing to activate against APCs bearing pathogenic-pMHC (see Text S1 and Figure S1 for different parameters).

**Figure 3:**
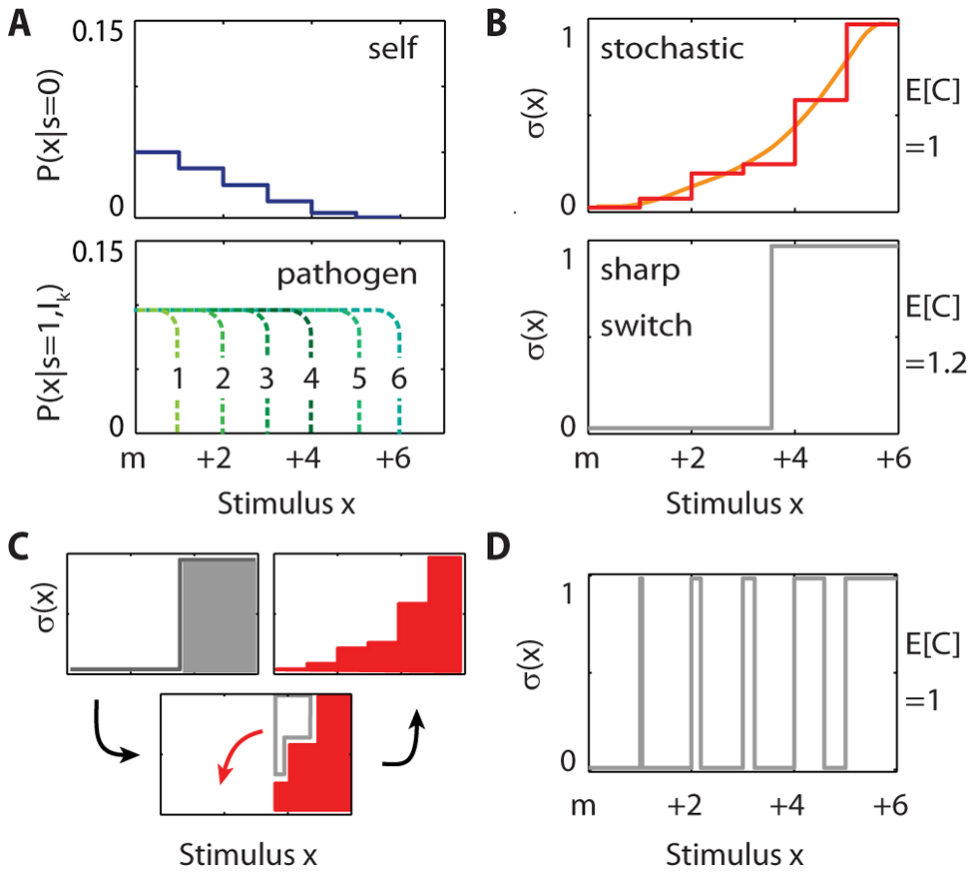
A simple model demonstrates that stochastic decisions can enable T cells to achieve complex goals with a simpler signaling network than that required for an optimal deterministic decision rule. (A) The probability distributions for the stimuli T cells receive from self (*P(x|s = 0)*, upper) and pathogenic (*P(x|s = 1,I_k_)*, lower) pMHC, where *I_k_* denotes the *k^th^* infection. For weak stimulus strengths, the probability densities are expected to be similar (and high) for self and pathogenic pMHC; *m* denotes an intermediate stimulus strength, above which the probability distributions are different. The numbers on the abscissa are in arbitrary units. The six possible infections (distributions of pathogenic stimuli) occur with probability 0.001, 0.049, 0.15, 0.25, 0.3, and 0.25, from *I_1_* to *I_6_*, so that infections which lead only to relatively weak stimuli are unlikely. Similarly, strong stimuli from self are unlikely. (B) For the probability and cost models in the main text, the best single sharp threshold (grey) has a higher expected cost (E[C]) than a stochastic decision rule (red). Reported E[C] is normalized by the expected cost of the stochastic decision rule. The stochastic decision rule is piecewise constant because the stimulus-strength probability distributions are discretized (see panel A). The orange curve helps visualize the stochastic solution. (C) The best stochastic decision rule (red) can be created from a sharp threshold (grey) by shifting immune pressure (red arrow) from strong stimuli to weaker stimuli. This shift helps balance the risk that some self pMHC lead to strong stimuli and some pathogens lead only to relatively weak stimuli. (D) A complex deterministic decision rule that alternates between never activating (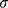 = 0) and always activating (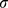 = 1) performs as well as the best stochastic one (panel B). Implementing this decision rule would require a complex signaling network.

The common environment is treated by choosing a probability model that satisfies Eq. 5. The probability model incorporates many possible infections, *I_k_*, each of which corresponds to a different environment, characterized by distributions of stimulus strengths *x­*_i_ in encounters between T cells and APCs bearing pathogenic-pMHC (*s­_i_ = 1*): *P(x_i_|s_i_ = 1,I_k_)* (Fig. 3A). Independent of the infection, APCs bearing only self pMHC (*s_i_* = 0) lead primarily to weak stimulus strengths *x_i_* as described by *P(x_i_|s_i_ = 0)* (Fig. 3A). (For very weak stimuli, the distributions of stimulus strengths from foreign- and self-pMHC are likely to be similar since negative selection does not affect the distributions of weak stimuli from either pathogenic or self pMHC; we assume T cells never activate for these stimuli and so do not include them in our model.) A simple probability model for the APC types **s**, the stimulus strengths **x**, and the decisions **d** is (consistent with Eq. 5):
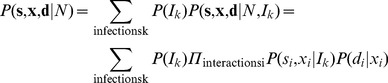
(13)


Eq. 13 weights the probability distributions for **s**, **x**, and **d** in each infection *I_k_* by the infection's probability, *P(I_k_)*, summed over all infections. The probability distribution for the APC types **s** and the stimulus strengths **x** during an infection, *P(s_i_,x_i_|I_k_)*, is determined by the distributions *P(x_i_|s_i_ = 0)* and *P(x_i_|s_i_ = 1,I_k_),* described above, and by the overall probabilities that an APC bears only self (*s_i_* = 0) or also pathogenic (*s_i_* = 1) pMHC during an infection. The probability that the infection confronted is the k^th^ one, *P(I_k_)*, is chosen so that it is unlikely that the immune system confronts an infection that leads only to weak stimulus strengths. We assume that the number of encounters during an infection is large enough for the T cell population to sample the probability distributions of stimulus strengths well. Given the model in Eqs. 12 and 13 with the specified parameter values and assumptions, the expected cost incurred to the host for any decision rule (Eq. 2) can be evaluated numerically (see Text S1). We find that a stochastic decision rule outperforms any deterministic sharp threshold that could be obtained by suppressing stochasticity (Fig. 3B).

A threshold stimulus strength sharply separating decisions to always activate and to never activate enforces all-or-nothing immune pressure over different regions of stimulus strength. This is unlikely to be the appropriate balance between the risk that some self-peptides will generate strong stimuli or that some infections will lead only to relatively weak stimuli (e.g. via immune evasion techniques) (Fig. 3C). Any infection which leads only to stimulus strengths weaker than the threshold will proliferate without inducing a T cell response. The T cell population could lower this risk by reducing the threshold for activation. However, this would produce a commensurate response against self-peptides that lead to stimulus strengths below, but close to, the threshold. A stochastic decision rule achieves a balance between the risks of autoimmunity and infection, critical for the host's survival, by ensuring some response to sub-threshold infections while not risking a full response against self-peptides.

Just including coupling between T cell decisions via the incurred costs and interactions in a simple way results in stochastic decisions being beneficial, suggesting that this would definitely be so if additional sources of coupling between T cells were included (e.g. coupling through cytokines), though these may provide new qualitative explanations in addition to the explanation in the previous paragraph. Even in our simple model a single free parameter – the location of the sharp threshold – does not give the T cell population enough flexibility to optimize its response. The increased degrees of freedom available with a stochastic response (how often to activate at each of many values of the stimulus) are required. It would be remarkable if adding further complexity to the model (e.g. more interactions among its components) decreased the number of degrees of freedom required to optimize the response. These arguments suggest that the conclusions are robust to specific features of the model.

### Stochastic responses are not necessary for diversification but enable complex functions with simpler signaling machinery

Could T cells obtain the same performance deterministically, albeit with a different signaling machinery or is stochasticity necessary for diversifying the response? We searched for deterministic decision rules, more complicated than a single sharp switch, which are as good as the optimal stochastic solution. The Dvoretzky-Wald-Wolfowitz (DWW) theorem suggests that it is always possible to find such a deterministic solution, for a model such as that described by Eqs. 12 and 13, as long as the probability distributions of stimuli observed by T cells are continuous [29], [30]. The latter should be true because two T cells are unlikely to see exactly the same stimulus due to abundant genetic (different TCRs) and environmental (different pathogens) diversity. By searching for optimal deterministic solutions that are not restricted to being a single sharp switch (see Text S1), we obtain a deterministic optimal decision rule (Fig 3D) that performs as well as the stochastic solution. The deterministic solution hedges the risk that an infection will lead to only weak stimuli, or that self peptides will lead to strong stimuli, by alternating between always activating and never activating as the stimulus strength increases from the lower end of the intermediate stimulus range to the upper end. Then, although one T cell may receive an intermediate stimulus that leads certainly to activation, other T cells will receive similar (but not identical) stimuli which lead certainly to remaining inactive. On average, the result is the same as the stochastic decision rule. (The deterministic solution in Fig. 3D is not unique for our model, but all solutions share the features we describe; see Text S1.)

The DWW theorem makes precise the intuition that, in contrast to isogenic, sensorless populations, stochasticity is not needed for diversification of the response when there is considerable genetic or environmental diversity to draw on, as for the T cell population. However, Fig. 3D shows that the optimal deterministic decision rule that exploits the environmental and genetic diversity is far more complicated than the relatively simple, single sharp threshold, requiring the intracellular signaling machinery to frequently switch between always activating and never activating over increasing intervals of the stimulus. These many sharp thresholds could only be implemented by a complex signaling network (e.g. many coordinated feedbacks; see Text S2 for an example signaling network). By making stochastic decisions, T cells can perform just as well with a far simpler signaling network (e.g. a single positive feedback for this part of the T cell response), which may be easier to control and evolve.

## Discussion

The role of stochasticity in biological decisions has been viewed in two ways. First, as a nuisance that is potentially costly to suppress (“noise”) [5]. Second, as a way for populations with limited environmental or genetic diversity to diversify their responses, which, in turn, optimizes some measure of performance (e.g. population growth in a varying environment) [9]. Many biological systems can utilize considerable environmental and genetic diversity to diversify their responses. In such systems, it would appear that stochasticity is unnecessary and potentially just “noise.” To understand the role of stochasticity in such systems, we studied general models motivated by T cell biology. The models suggest that a population's responses can indeed be diversified without stochasticity, using deterministic signaling machinery, if no two cells are exactly the same or receive exactly the same stimulus. However, when cells in a population are strongly coupled to each other, the signaling machinery that would be required to diversify deterministically is exceedingly complex. With stochastic responses, we find that populations like T cells can achieve optimal diversification with relatively simple signaling machinery.

This role for stochasticity is particularly important as experiments reveal stochastic decision-making in ever more complex mammalian cellular responses, like apoptosis decisions and NF-κB responses to TNF-α [3], [4]. Not only can mammalian populations utilize environmental diversity to diversify their responses, they, like T cells, can utilize genetic (or epigenetic) diversity, since they coexist in the host to perform complex functions. In this respect, they differ from canonical bacterial systems that cannot utilize genetic diversity because different genotypes compete evolutionarily.

Previous results, focused on the role of stochasticity in diversification, have shown that stochastic responses optimize the performance of certain systems in terms of an expected cost. Here we have demonstrated a role for stochastic responses, in systems utilizing environmental and genetic diversity, even though they are not necessary to minimize the expected cost.

In populations that are only weakly coupled, we find that relatively simple signaling machinery can diversify the response deterministically. In such populations, stochastic responses may merely be “noise.”

Synthetic cellular signaling networks have been successfully constructed in bacterial systems to test theoretical predictions in biological systems (Bashor et al. 2010). These networks provide a potential route for understanding how coupling between different decision-makers in biological systems, under the conditions described in this manuscript, can lead to stochastic decision making, by engineering different types of coupling within bacterial populations in evolutionary studies. In addition, bacterial systems that can switch among different phenotypes can be used to test the prediction that the deterministic decision rule in Fig. 3D is difficult to implement with signaling machinery. Bacterial populations can be presented sequentially with different concentrations of a harmless chemical that they are able to sense; if the concentration of the harmless chemical falls within one of the ranges in Fig. 3D such that σ(x) = 1, a chemical that is toxic to the high-growth phenotypic state, but which the bacteria are unable to independently sense, is also presented. To grow optimally in this experiment, bacteria must evolve a phenotype-switching strategy according to the decision rule in Fig. 3D; a stochastic strategy is not equivalent. Thus, after allowing bacterial evolution, the experiment would reveal how successfully bacteria can evolve complicated decision rules like the one in Fig. 3D.

An implication of our results for T cell biology is that to understand the design of an individual T cell's signaling network it is necessary to analyze the behavior of the T cell population. Therefore, experimental studies to understand stochasticity in T cell signaling machinery will require studying the immune system in a systemic fashion in whole hosts subject to multiple infections.

## Supporting Information

Figure S1
**Varying the cost function and probability distributions does not change the qualitative results in the main text.** (A) An alternate model for the probability distributions for the stimuli T cells receive from self (*P(x|s = 0)*, upper) and pathogenic (*P(x|s = 1,I_k_)*, lower) pMHC, where *I_k_* denotes the *k^th^* infection. For weak stimulus strengths, these probability distributions are expected to be similar for self and pathogenic pMHC with high values for *P*; *m* denotes an intermediate stimulus strength, above which these probability distributions are different. The numbers on the abscissa are in arbitrary units. The six possible infections (distributions of pathogenic stimuli) occur with probability 0.001, 0.099, 0.2, 0.2, 0.25, and 0.25, from *I_1_* to *I_6_*, so that infections which lead only to relatively weak stimuli are unlikely. Similarly, strong stimuli from self are unlikely. (B) For the probability and cost models, the best single sharp threshold (grey) has a higher expected cost (E[C]) than a stochastic decision rule (red). Reported E[C] is normalized by the expected cost of the stochastic decision rule. The optimal decision rules reflect the discretization of the probability distributions describing stimulus strengths (see panel A). A complex deterministic decision rule that alternates between never activating (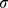 = 0) and always activating (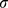 = 1) performs as well as the best stochastic one. Implementing this decision rule would require a complex signaling network.(TIF)

Text S1 **Simple model of the T cell population.**
(PDF)

Text S2 **Implementing deterministic decision rules with many sharp thresholds.**
(PDF)
