# Supplementary figures and images for: Stochastic Responses May Allow Genetically Diverse Cell Populations to Optimize Performance with Simpler Signaling Networks
Source: PLoS One. 2013 Aug 7;8(8):e65086. doi: 10.1371/journal.pone.0065086 (PMC3737226; doi:10.1371/journal.pone.0065086)

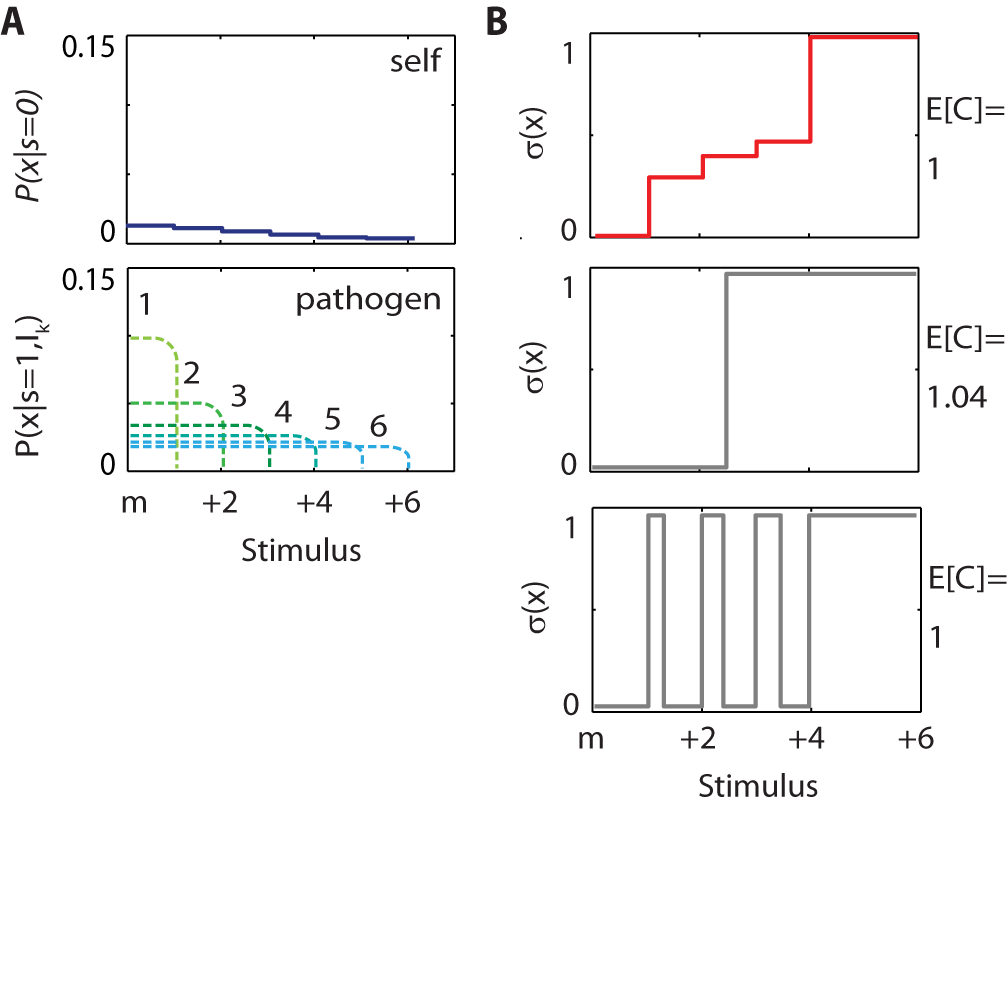

Supplement: Figure S1 — Varying the cost function and probability distributions does not change the qualitative results in the main text. (A) An alternate model for the probability distributions for the stimuli T cells receive from self (P(x|s = 0), upper) and pathogenic (P(x|s = 1,Ik), lower) pMHC, where Ik denotes the kth infection. For weak stimulus strengths, these probability distributions are expected to be similar for self and pathogenic pMHC with high values for P; m denotes an intermediate stimulus strength, above which these probability distributions are different. The numbers on the abscissa are in arbitrary units. The six possible infections (distributions of pathogenic stimuli) occur with probability 0.001, 0.099, 0.2, 0.2, 0.25, and 0.25, from I1 to I6, so that infections which lead only to relatively weak stimuli are unlikely. Similarly, strong stimuli from self are unlikely. (B) For the probability and cost models, the best single sharp threshold (grey) has a higher expected cost (E[C]) than a stochastic decision rule (red). Reported E[C] is normalized by the expected cost of the stochastic decision rule. The optimal decision rules reflect the discretization of the probability distributions describing stimulus strengths (see panel A). A complex deterministic decision rule that alternates between never activating ( = 0) and always activating ( = 1) performs as well as the best stochastic one. Implementing this decision rule would require a complex signaling network. (TIF) [file pone.0065086.s001.tif]
