# Supplementary material for: Stochastic Responses May Allow Genetically Diverse Cell Populations to Optimize Performance with Simpler Signaling Networks
Source: PLoS One. 2013 Aug 7;8(8):e65086. doi: 10.1371/journal.pone.0065086 (PMC3737226; doi:10.1371/journal.pone.0065086)
Supplement: Text S1 — Simple model of the T cell population. (PDF) [file pone.0065086.s002.pdf]

Supplemental Text

S1. Simple model of the T cell population

S1A. Variations of the simple T cell signaling model

In the main text, our simple model consisted of the cost function in Eq. 9, with c_1=50, c_2=0.2, and c_3=40, and the probability model in Eq. 10 with distributions in Fig. 3A. (These parameters weight the cumulative mistakes against self and pathogenic pMHC roughly equally.) In this section, we show that a different cost function with different probability distributions leads to the same qualitative results as the example in the main text, suggesting the results do not particularly depend on the choices of these model inputs. The cost function and probability model presented here have the same qualitative properties motivated in the main text. The cost function is:

$$C(\vec{e},\vec{d}) = e^{-c_1 f_1(\vec{e})} + c_2\left(e^{c_3 f_0(\vec{e})} - 1\right) \tag{S8}$$

c_1=35

c_2 =0.1

c_3=150

The probability distributions for encounters with self and pathogenic pMHC are presented in Fig. S1A. As in the main text, we model only an intermediate range of stimulus, since it is assumed T cells will not activate at very weak stimulus.

As with the model in the main text, the best stochastic solution outperforms the best single sharp threshold (Fig S1B). The percentage change is small, but suffices to confirm that stochastic decisions outperform single sharp thresholds. (Because of the simplifications, the model is not quantitative.)

S1A. Optimization of the simple T cell model

In the main text, we considered a simple model in which the host encounters a single infection. Which particular infection the host encounters is uncertain. The cost function in Eq. 9 and probability model in Eq. 10 set up an optimization problem for the decision rule. To simplify the calculation, we made the assumption in the main text that the number of encounters in each infection is large enough so that, within a particular

infection, the distributions of stimuli from self and pathogenic pMHC are well-sampled. In the main text, we introduced the notation f_0 and f_1 for the fractions of encounters with self and pathogenic pMHC that activate T cells. When the distributions are well sampled, the fractions f_0 and f_1 converge to probabilities:

$$f_0(\vec{e}) \rightarrow \int dx P(x \mid s=0)\sigma(x) \quad \text{(S6)}$$

$$f_1(\vec{e}) \rightarrow \int dx P(x \mid s=1, I_k)\sigma(x)$$

That is, the probability a T cell activates in an encounter with self pMHC is just the probability the T cell activates given the stimulus x ($\sigma(x)$) times the probability the stimulus is actually x in an encounter with self pMHC ($P(x|s=0)$), integrated over all possible stimuli x; Eq. S6b follows similarly. Then, the only uncertainty in the expectation in Eq. 1 is which particular infection the immune system confronts (out of 6), since the many values of $\vec{s}$, $\vec{x}$, and $\vec{d}$ that might be encountered during the infection are now integrated out in Eq. S6. Note that, because the optimization depends separately on f_0 and f_1, the relative probability of pMHC being self or pathogenic ($P(s=0)$ vs. $P(s=1)$) in Eq. 10 is irrelevant.

The simple probability model we have chosen is constant over unit intervals of the stimulus (Fig. 3A), in order to simplify computation of the optimal decision rule. As a result, the optimization problem can be transformed from a functional optimization over all σ to an optimization over vectors $\vec{v}$ where:

$$v_i = \int_{\text{ith unit interval}} \sigma(x)dx \quad \text{(S7)}$$

Each v_i is constrained to be between 0 and 1, inclusive (because each interval is of unit length and the decision rule falls between 0 and 1, inclusive, for all x).

If more than one element of the optimal solution $\vec{v}$ is not strictly 0 or 1, then a stochastic strategy is strictly better than a single sharp threshold, since sharp thresholds have v_i equal to 0 or 1 (no or complete activation) on all intervals except the one the threshold falls in.

In general, the optimum decision rule σ^* corresponding to the optimal solution $\vec{v}$ is degenerate, since Eq. S7 is not invertible. The stochastic decision rule plotted in Fig. 3B was obtained by letting σ(x) be constant over each interval. The deterministic

decision rule plotted in Fig. 3D was obtained by taking σ(x)=1 over the first part of each interval, and then σ(x)=0 over the second part of each interval, such that the appropriate value for v_i was obtained. Though slightly simpler deterministic decision rules can be found by varying the choice of σ(x), they are still more complicated than the stochastic decision rule or a single sharp threshold. The best single sharp threshold (Fig. 3B) was obtained by explicitly searching over all possible threshold locations.
