# Supplementary material for: Stochastic Responses May Allow Genetically Diverse Cell Populations to Optimize Performance with Simpler Signaling Networks
Source: PLoS One. 2013 Aug 7;8(8):e65086. doi: 10.1371/journal.pone.0065086 (PMC3737226; doi:10.1371/journal.pone.0065086)
Supplement: Text S2 — Implementing deterministic decision rules with many sharp thresholds. (PDF) [file pone.0065086.s003.pdf]

Supplemental Text

S2. Implementing deterministic decision rules with many sharp thresholds

The following network topology is capable of producing a deterministic decision rule with many sharp thresholds, as in Fig. 3D. An analogue, graded signal is sent in parallel to two downstream signaling modules, module A and module B. Both modules produce sharp-threshold responses based on the analogue input, activating only when the input exceeds a certain threshold, but the two modules have different values of the threshold, x_A and x_B, respectively. For example, the modules both consist of positive-feedback loops, but with different values of the kinetic parameters so that the thresholds are different. Without loss of generality, assume that the threshold for module A is lower than the threshold for module B, $x_A < x_B$. The output of the two modules is merged in a downstream module, which operates as an exclusive-or gate: it activates only when module A, but not module B is active or when module B is active, but not module A. (By construction, since $x_A < x_B$, only the first of these possibilities can occur.) Such exclusive-or gates can be constructed from molecular components [1,2]. The output of this final step will be a nonmonotonic output function that never activates for stimuli less than x_A, activates for simuli between x_A and x_B, and never activates for stimuli greater than x_B. By adding more modules in parallel and further logical gates to process the outputs, deterministic decision rules like the one in Fig. 3D can be constructed in principle out of molecular components. Thus, many coordinated positive feedback loops can produce decision rules such as the one in Fig. 3D.

1. de Ronde W, ten Wolde PR, Mugler A (2012) Protein Logic. Biophysical Journal 103: 1097-1107.
2. Arkin AP, Ross J (1994) Computational functions in biochemical reaction networks. Biophysical Journal 67: 560-578.
